# Case report: A vertebral bone spur as an etiology for spinal cord herniation: case presentation, surgical technique, and review of the literature

**DOI:** 10.3389/fsurg.2023.1238448

**Published:** 2023-08-08

**Authors:** S. Hunziker, A. Örgel, M. Tatagiba, S. D. Adib

**Affiliations:** ^1^ Department of Neurosurgery, University of Tuebingen, Tübingen, Germany; ^2^ Department of Diagnostic and Interventional Neuroradiology, University of Tuebingen, Tübingen, Germany

**Keywords:** spinal cord herniation, osteophyte, ISCH, Brown-Sequard syndrome, bone spur, dural patch

## Abstract

**Objective:**

The pathophysiology of idiopathic spinal cord herniation remains unknown. However, several different factors have been postulated, such as congenital causes (ventral dura mater duplication, preexisting pseudomeningocele, or other congenital dural defects), inflammation, remote spinal trauma, or thoracic disc herniation. Herein, the diagnosis and surgical treatment of a patient with spinal cord herniation caused by an intraspinal bone spur is presented along with a relevant literature review.

**Case presentation:**

A 56-year-old male patient presented with a non-traumatic Brown-Sequard syndrome persisting for over 1 year. A magnetic resonance imaging of the spinal axis revealed a ventral spinal cord displacement in the level of T 6/7. A supplementary thin-sliced computed tomography of the spine revealed a bone spur at the same level. For neurosurgical intervention, T 6 and T 7 laminectomy was performed. The cranial and caudal end of the right paramedian ventral dural defect was visualized and enlarged. Following extradural spinal cord mobilization by denticulate ligament transection, the spinal cord was finally released. The spinal cord was rotated and the ventral closure of the dural defect was performed by continuous suture. The patient recovered from surgery without additional deficits. The patient’s postoperative gait, sensory, and motor function deficits improved, and further neurological deterioration was prevented.

**Conclusion:**

Since the first description of spinal cord herniation by Wortzman et al. in 1974, approximately 260 cases have been reported in the literature. In addition to other causes, intraspinal bone spur is a possible cause of spinal cord herniation.

## Introduction

Ventral spinal cord herniation was first described in 1974 by Wortzman et al., when a herniated spinal cord was incidentally noticed during a thoracotomy operation for disc herniation (1). Since then, more than 246 cases have been reported in the literature (2). Due to this increased reporting of the disorder, a sufficient level of awareness has been created among clinicians (3).

The pathomechanism of this disorder is a ventral displacement of the spinal cord across a defect in the dura, leading to ventral adhesion to the vertebra. Because of the constriction of the spinal cord, blood flow to the herniated part decreases gradually, which leads to progressive ischemic myelopathy and neurological impairment. Symptoms may manifest before a herniation can be detected on magnetic resonance imaging (MRI). Idiopathic spinal cord herniation (ISCH) generally occurs in the upper thoracic spine, most often with the involvement of the T 4—T 8 level (4). Also most often, herniation occurs anteriorly to the spinal cord, with only two cases reported of a herniation that occurred posteriorly (5, 6).

A total of five patients (mean age: 66.2 ± 5.97 years) with spinal cord herniation underwent surgery at the Department of Neurosurgery of the University of Tuebingen between January 2015 and May 2023. Out of these five, we found one patient with spinal cord herniation associated with an intraspinal bone spur (also referred to as osteophyte). This finding of an intraspinal bone spur is in line with only a few previously reported cases (7–10).

## Case presentation

A 56-year-old male patient suffered from a progressive myelopathy with features of the Brown-Sequard syndrome, which persisted for over a year. A neurological examination revealed an ataxic gait, limited walking distance, monoparesis (M4) of the right leg flexor, increased reflexes of the lower right extremity, and hypoesthesia for pain, touch, and temperature of the left leg and up to a level of T 7. Babinski sign was negative. The patient did not have any history of trauma, central nervous system inflammation, or spinal manipulation.

**Figure 1:**
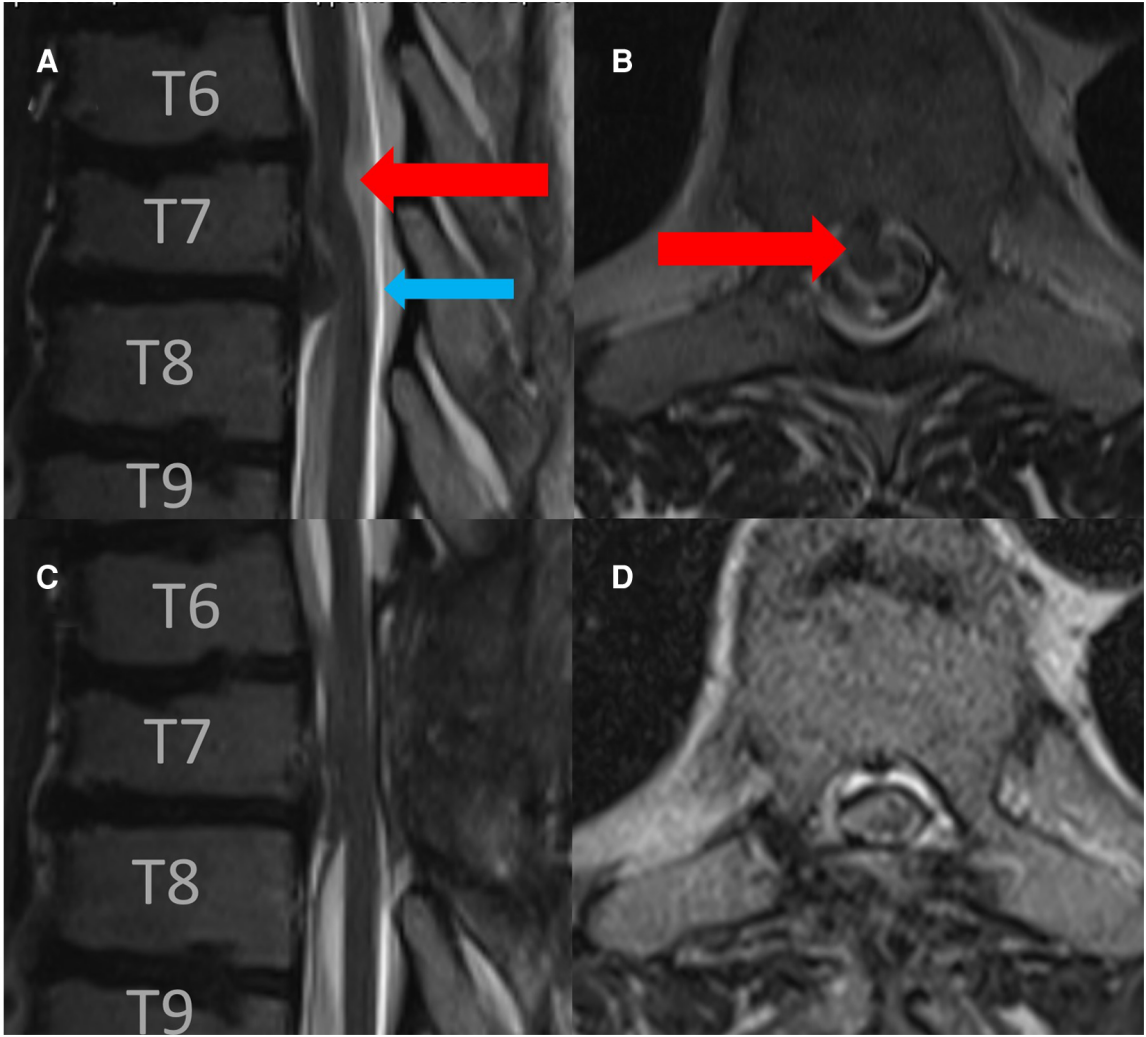
Preoperative sagittal (**A**) and axial (**B**) T2-weigthed MRI revealed a spinal cord herniation at the level of T 6/7 (read arrow) and disc herniation at the level of T 7/8 (blue arrow). Postoperative sagittal (**C**) and axial (**D**) follow-up T2-weigthed MRI after 3 months revealed the release of spinal cord herniation.

**Figure 2:**
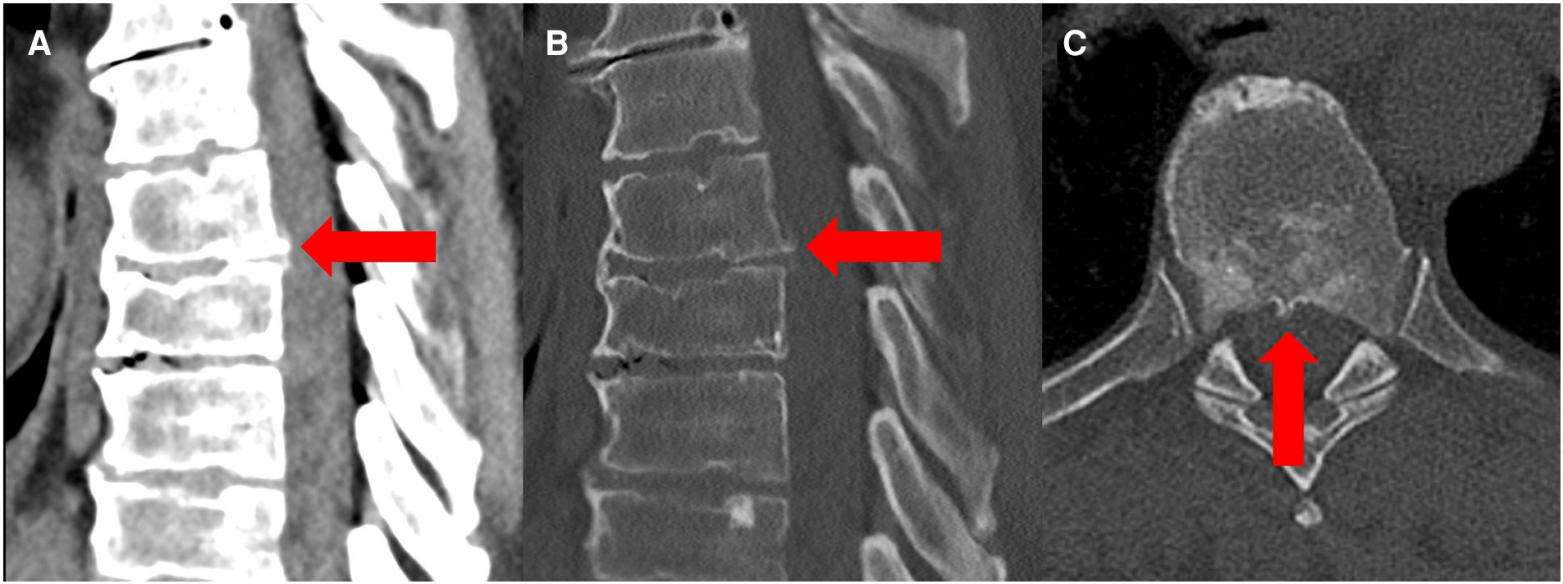
Preoperative sagittal and axial CT scan in a soft tissue window (**A**) and bone window (**B,C**) revealed a bone spur at the level T6/7 (red arrow) as a possible cause of spinal cord herniation.

A magnetic resonance imaging of the spine revealed spinal cord displacement to the ventral location of the spinal canal at the level of T 6/7, a possible adhesion of the cord tissue to the upper part of the T 7 dorsal vertebral body, and a hyperintensity of the spinal cord in T2-weighted imaging at the same level (Figures 1A,B). In addition, disc protrusions at levels T4/5, T 7/8, and T 10/11 were present with a slight compression of the spinal cord at level T 7/8, which was below the spinal cord herniation. A supplementary thin-sliced computed tomography of the spine revealed a bone spur at the level of T 6/7 (Figure 2). Because of the progressive neurological deficits and correlated radiological findings, the option of surgical repair of the spinal cord herniation (laminectomy of T 6 and T 7, microsurgical release of the herniated spinal cord, and closure of the ventral dural defect) was discussed with the patient.

### Intervention

**Figure 3:**
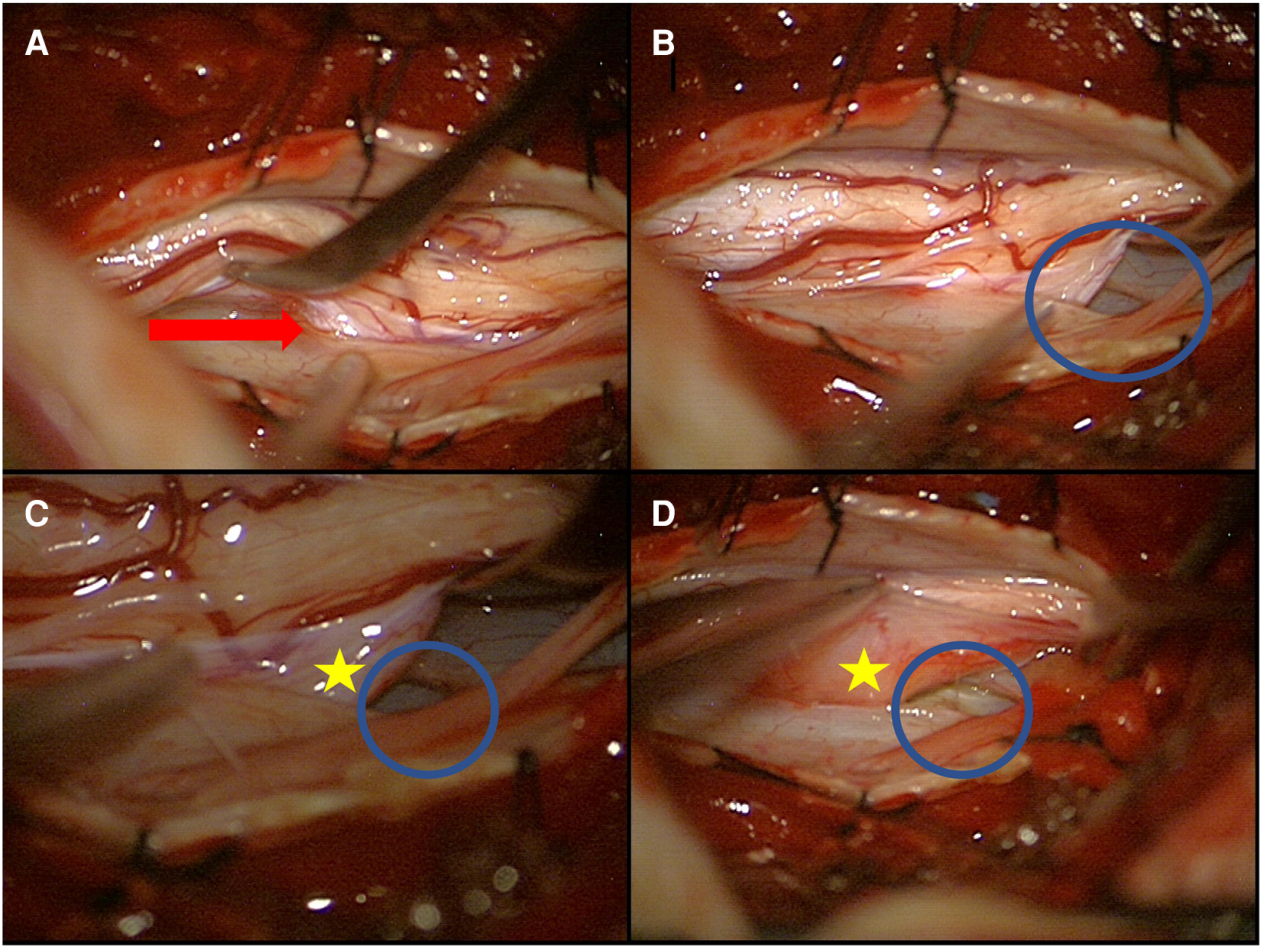
Intraoperative image of an anterior dural perforation with spinal cord herniation (**A**) and a lateralized spinal cord (**B–D**) (red arrow: spinal cord herniation; blue circle: dural defect; yellow star: extradural part of the spinal cord).

The patient was placed in a prone position under general anesthesia with intraoperative monitoring (IOM) including motor-evoked potentials (MEPs) and somatosensory-evoked potentials (SSEPs) of the lower extremities. High-dose methylprednisolone succinate (Urbason®) administration for treatment of myelopathy was given intravenously according to the Urbason scheme (as a bolus of 30 mg/kg of body weight, followed by infusion at 5.4 mg/kg/h for 23 h). Localizing fluoroscopy was performed to identify the level of interest. Then, a thoracic laminectomy of T 6 and T 7 was performed in standard fashion. Intraoperatively, the cranial and caudal ends of the right paramedian ventral dural defect were visualized (Figure 3). With the microsurgical technique, the denticulate ligaments were transected bilaterally to allow sufficient mobilization of the spinal cord. The dural defect was enlarged to mobilize the spinal cord adequately. The spinal cord was released carefully from the entrapment and adhesion. The released spinal cord was rotated and repositioned intradurally. The herniated disk fragment and the bone spur were carefully resected from the intra- and extradural spaces. Then, the ventral dural defect was closed by continuous suture. Glue was applied over the suture. MEPs, SSEPs, and D-waves did not get altered during the surgery.

### Postoperative Course and follow-up

Postoperatively, the patient exhibited no new neurological deficits. In contrast, postoperatively, his gait dysfunction improved and his sensory, motor deficits were significantly alleviated. In the follow-up after 3 months, the magnetic resonance image of the spinal axis showed good release of the spinal cord without reattachment (Figures 1C,D).

## Discussion

To date, many different pathophysiological factors have been postulated as a potential etiology for spinal cord herniation. However, no specific etiologic factor has been found.

One factor that has been suggested is congenital malformation (11, 12). What is critical to this suggestion is that there is delay in the occurrence of symptomatology; it occurs only during one’s middle age. However, the exact reason for the delayed occurrence of herniation is inadequately accounted for at present. One proposition (13) is that symptomatology might predate herniation, which accounts for the fact that a structural abnormality already exists at the herniation level; however, it does not explain why herniation *per se* should be delayed (14).

Previous studies have demonstrated that a congenital duplication of the ventral dura allows herniation of the spinal cord through the inner dura, which is possibly attributable to a neurenteric canal remnant. The spinal cord herniates within this defect and becomes strangulated, causing neurological deficits; this has been reported by Tyagi et al. (15).

According to Najjar et al. (16), it seems to be an acquired condition possibly caused by an inflammatory process, which results in adherence between the spinal cord and the dura, with erosion, formation of a dural defect, and then herniation occurring with cerebral spinal fluid (CSF) pulsations.

Reports about traumatic events associated with spinal cord herniation have also been published (17, 18). However, one must be careful not to attribute trauma as the scientific cause of the dural defect if no injury is reported at the relevant level (19). Often times, there is spontaneous cord herniation with a history of irrelevant or trivial trauma not differentiated from actual post-traumatic spinal cord herniation (18). In case of fractured vertebra, there is a sharp dural tear with herniation at the same level due to a fractured bone (20) or a penetrating (i.e., stab) injury (21) at the level of herniation, which may be legitimate post-traumatic cord herniation. This etiology may also be identified by its characteristics, which differ from those of non-traumatic spinal cord herniation. In traumatic spinal cord herniation, the level of herniation is not typically mid-thoracic ventral occurring mainly in middle age, but spinal cord herniation occurs at the level of trauma, which may be cervical or low-thoracic. Herniation may also occur at the dorsal level instead of at the ventral level, may be associated with nerve root avulsion, and occur at any age (22, 23).

Intraoperatively, an enlarged posterior arachnoid space can also be identified in a majority of patients which has to be differentiated from posterior arachnoid cyst (2, 3). Rather it is a co-occurrence due to the changed anatomy of the spinal cord with a reduced caliber and an increased amount of posterior intraspinal space.

ISCH almost always occurs at the thoracic level; this may be attributed to the anatomical characteristics of the thoracic spinal cord, with its typical kyphotic curve leading to the ventral position of the spinal cord in relation to the cervical and lumbar regions. During the performance of daily activities, the flexion and extension of the thoracic spine may lead to dural injury when there is an associated vertebral bony spur. It may lead to unrecognized trauma to the ventral dura mater and further dural laceration and spinal cord herniation. Dorsal thoracic dural defect with posterior spinal cord herniation has been reported only twice so far. Dorsal spinal cord herniation causing a perforation of the posterior elements has also been reported (5, 6).

The spinal cord develops adhesions to the dural defect and gradually becomes strangled, which, in turn, results in strangulation of the spinal tracts. The consequent tethering/distortion and ischemia of the neural elements lead to neurological deficits such as Brown-Sequard syndrome and paraparesis. A diagnosis of spinal cord herniation requires that the patient undergo an MRI of the spine. For symptomatic patients, operative retrieval of the herniated spinal cord and repair of the dural defect are recommended. Two different techniques are employed: one is direct closure of the defect by primary suturing and the second is placement of an artificial dural graft in some patients, which is accompanied by an enlargement of the dural defect to facilitate cord retraction. Widening of the dural defect is significantly associated with postoperative improved neurological functioning (2). Intraoperative ultrasound may be helpful in detecting the location of the herniation (24, 25).

In addition to the few previously reported cases (7–10), our case shows that intraspinal spur and disc herniation may be a cause of spinal cord herniation (similar to the pathophysiology of intracranial hypotension). Bone spur occurs exactly at the spinal cord herniation level, which is highly suggestive of the origin of the herniation. Mechanic stress caused by bone spur may lead to dural defect, followed by a herniation of the spinal cord through this defect. CSF leakage in the thoracic epidural space following dural laceration leads to CSF leakage with intracranial hypotension and position-dependent headache (9, 10). In 28.9% of ISCH patients, an extradural fluid can be detected (26). As a consequence, a decreased pressure in the thoracic epidural space facilitates the herniation process. In our patient, below the level of the herniation, at T 7/8, there was disc protrusion, which could lead to altered CSF flow and enhance herniation through a dural tear. Intraoperatively, at the spinal cord herniation level, a disc protrusion was identified, which was not very visible on preoperative MRI probably because of an anterior herniation of the spinal cord, subsequently, it was resected.

Tight dural closure is important for preventing dural leakage postoperatively. Otherwise, there is a risk for the development of intracranial hypotension and consequent subdural hematoma with neurological deterioration (27).

Brus-Ramer and Dillon (26) analyzed 70 published cases of patients with spinal cord herniation with associated disc protrusion and accompanying osteophytes. This study showed that in 67.1% of patients, herniation occurred at the disc level. In 30.2% of patients, there was herniated nucleus pulposus, and in 30.2%, osteophytes occurred. Because of this high percentage of associated nucleus pulposus herniation and osteophytes, it may be concluded that this factor plays a role in the pathogenesis of spinal cord herniation.

### Surgical outcome

Groen et al. (2) concluded in a meta-analysis that 71% of 121 ISCH patients showed neurological improvement after surgery, while 20% showed no change, and 7% experienced a neurological decline. Similar results were obtained by Summers et al. (28).

Hirose et al. (29) concluded that there was no significant difference in the level of mean recovery among patients with different locations (ventrolateral vs. ventral) of the dural defect, but they found that there was a significant correlation between age at the time of surgery and recovery rate and also between disease duration and recovery rate.

## Conclusion

In this study, we reported a case of a patient with spinal cord herniation at the thoracic level, which was most probably attributed to a vertebral bone spur–induced dural tear and associated disc protrusion at the herniation level. Because of the kyphotic structure of the spine, sheering force exerted on the dura by movement, finally leading to dural rupture. Consecutive CSF leakage may result in intracranial hypotension. Because of altered CSF flow, the spinal cord may finally herniate through the defect in the dura. Strangulation of the cord leads to spinal cord impairment and clinical Brown-Sequard syndrome or spastic paralysis.

In addition to previously reported cases, our case supports the theory that intraspinal bone spur may cause spinal cord herniation and intracranial hypotension because of CSF leakage.

## Data Availability

The original contributions presented in the study are included in the article/supplementary material, further inquiries can be directed to the corresponding author.

## References

[1] Wortzman G Tasker RR Rewcastle NB Richardson JC Pearson FG. Spontaneous incarcerated herniation of the spinal cord into a vertebral body: a unique cause of paraplegia. Case report. J Neurosurg. (1974) 41:631–5. 10.3171/jns.1974.41.5.0631 4424434

[2] Groen RJM Lukassen JNM Boer GJ Vergeer RA Coppes MH Drost G Anterior thoracic spinal cord herniation: surgical treatment and postoperative course. An individual participant data meta-analysis of 246 cases. World Neurosurg. (2019) 123:453–63.e15. 10.1016/j.wneu.2018.11.229 30529595

[3] Berg-Johnsen J Ilstad E Kolstad F Züchner M Sundseth J. Idiopathic ventral spinal cord herniation: an increasingly recognized cause of thoracic myelopathy. J Cent Nerv Syst Dis. (2014) 6:85–91. 10.4137/JCNSD.S16180 25336997 PMC4196882

[4] Runza G Maffei E Cademartiri F. Idiopathic herniation of the thoracic spinal cord: an often mis-diagnosed clinical entity. Acta Biomedica Atenei Parmensis. (2021) 92:e2021143. 10.23750/abm.v92iS1.9947 PMC8142771 33944828

[5] Le TC Grunch BH Karikari IO Mehta AI Owens TR Gottfried ON Dorsal thoracic spinal cord herniation: report of an unusual case and review of the literature. Spine J. (2012) 12:e9–12. 10.1016/j.spinee.2012.09.039 23092719

[6] Funao H Nakamura S Daimon K Isogai N Sasao Y Nishiyama M Idiopathic dorsal spinal cord herniation perforating the lamina: a case report and review of the literature. Acta Neurochir (Wien). (2021) 163:2313–8. 10.1007/s00701-021-04804-4 33745029

[7] Imai T Nakane Y Tachibana E Ogura K. Spinal cord herniation with characteristic bone change: a case report. Nagoya J Med Sci. (2015) 77:515–20. 26412899 PMC4574340

[8] Nakagawa H Kamimura M Uchiyama S Takahara K Itsubo T Miyasaka T. Idiopathic spinal cord herniation associated with a large erosive bone defect: a case report and review of the literature. J Spinal Disord Tech. (2003) 16:299–305. 10.1097/00024720-200306000-00013 12792347

[9] Kewlani B Garton ALA Hussain I Chazen JL Robbins MS Baaj AA Intracranial hypotension due to ventral thoracic dural tear secondary to osteophyte complex: resolution after transdural thoracic microdiscectomy with dural repair. Illustrative case. J Neurosurg Case Lessons. (2022) 3:CASE21615. 10.3171/CASE21615 36273860 PMC9379770

[10] Vishteh AG Schievink WI Baskin JJ Sonntag VK. Cervical bone spur presenting with spontaneous intracranial hypotension. Case report. J Neurosurg. (1998) 89:483–4. 10.3171/jns.1998.89.3.0483 9724127

[11] Bartels RHMA Brunner H Hosman A van Alfen N Grotenhuis JA. The pathogenesis of ventral idiopathic herniation of the spinal cord: a hypothesis based on the review of the literature. Front Neurol. (2017) 8:476. 10.3389/fneur.2017.00476 28955299 PMC5601982

[12] Morokoff AP Tress BM Kaye AH. Idiopathic spinal cord herniation. J Clin Neurosci. (2001) 8:180–3. 10.1054/jocn.2000.0819 11243773

[13] Ewald C Kühne D Hassler WE. Progressive spontaneous herniation of the thoracic spinal cord: case report. Neurosurgery. (2000) 46:493–5; discussion 495–6. 10.1097/00006123-200002000-00046 10690741

[14] Marshman LA Hardwidge C Ford-Dunn SC Olney JS. Idiopathic spinal cord herniation: case report and review of the literature. Neurosurgery. (1999) 44:1129–33. 10.1097/00006123-199905000-00112 10232549

[15] Tyagi G Prabhuraj AR Bhat DI Rao MB Devi BI. Duplication of ventral dura as a cause of ventral herniation of spinal cord—a report of two cases and review of the literature. World Neurosurg. (2019) 126:346–53. 10.1016/j.wneu.2019.02.143 30851464

[16] Najjar MW Baeesa SS Lingawi SS. Idiopathic spinal cord herniation: a new theory of pathogenesis. Surg Neurol. (2004) 62:161–70; discussion 170–1. 10.1016/j.surneu.2003.10.030 15261515

[17] Dix JE Griffitt W Yates C Johnson B. Spontaneous thoracic spinal cord herniation through an anterior dural defect. AJNR Am J Neuroradiol. (1998) 19:1345–8. 9726480 PMC8332207

[18] Watters MR Stears JC Osborn AG Turner GE Burton BS Lillehei K Transdural spinal cord herniation: imaging and clinical spectra. AJNR Am J Neuroradiol. (1998) 19:1337–44. 9726479 PMC8332200

[19] Tekkök IH. Spinal cord herniation–which one is really traumatic? AJNR Am J Neuroradiol. (2000) 21:609–12. PMC8174995 10730666

[20] Baur A Stäbler A Psenner K Hamburger C Reiser M. Imaging findings in patients with ventral dural defects and herniation of neural tissue. Eur Radiol. (1997) 7:1259–63. 10.1007/s003300050286 9377512

[21] Lee ST Lui TN Jeng CM. Spinal cord herniation after stabbing injury. Br J Neurosurg. (1997) 11:84–6. 10.1080/02688699746780 9156027

[22] Khattar NK Donovan AM Oxford BG Adams SWC Altstadt TJ. Traumatic ventral cervical spinal cord herniation: a case report. Cureus. (2019) 11:e4070. 10.7759/cureus.4070 31016097 PMC6464141

[23] Ijiri K Hida K Yano S Komiya S Iwasaki Y. Traumatic spinal-cord herniation associated with pseudomeningocele after lower-thoracic nerve-root avulsion. Spinal Cord. (2009) 47:829–31. 10.1038/sc.2009.38 19350043

[24] Ganau M Syrmos N Martin AR Jiang F Fehlings MG. Intraoperative ultrasound in spine surgery: history, current applications, future developments. Quant Imaging Med Surg. (2018) 8:261–7. 10.21037/qims.2018.04.02 29774179 PMC5941206

[25] Hawasli AH Ray WZ Wright NM. Symptomatic thoracic spinal cord herniation: case series and technical report. Oper Neurosurg. (2014) 10:E498–504. 10.1227/NEU.0000000000000437 PMC4134727 24871148

[26] Brus-Ramer M Dillon WP. Idiopathic thoracic spinal cord herniation: retrospective analysis supporting a mechanism of diskogenic dural injury and subsequent tamponade. AJNR Am J Neuroradiol. (2012) 33:52–6. 10.3174/ajnr.A2730 22158920 PMC7966180

[27] Barrenechea IJ Lesser JB Gidekel AL Turjanski L Perin NI. Diagnosis and treatment of spinal cord herniation: a combined experience. J Neurosurg Spine. (2006) 5:294–302. 10.3171/spi.2006.5.4.294 17048765

[28] Summers J Balasubramani Y Chan P Rosenfeld J. Idiopathic spinal cord herniation: clinical review and report of three cases. Asian J Neurosurg. (2013) 8:97–105. 10.4103/1793-5482.116386 24049553 PMC3775190

[29] Hirose Y Nagoshi N Tsuji O Kono H Iida T Suzuki S Natural history and surgical outcomes of idiopathic spinal cord herniation. Spinal Cord. (2023). 10.1038/s41393-023-00904-3. [Epub ahead of print] 37380759

